# Impact of COVID-19 on Public Interest in Breast Cancer Screening and Related Symptoms: Google Trends Analysis

**DOI:** 10.2196/39105

**Published:** 2023-06-06

**Authors:** Si Ying Tan, Matilda Swee Sun Tang, Chin-Ann Johnny Ong, Veronique Kiak Mien Tan, Nicholas Brian Shannon

**Affiliations:** 1 Department of Breast Surgery Division of Surgery and Surgical Oncology Singapore General Hospital Singapore Singapore; 2 Department of Breast Surgery Division of Surgery and Surgical Oncology National Cancer Centre Singapore Singapore Singapore; 3 Monash Health Melbourne, Victoria Australia; 4 Department of Sarcoma Peritoneal and Rare Tumours Division of Surgery and Surgical Oncology National Cancer Centre Singapore Singapore Singapore

**Keywords:** breast cancer screening, breast cancer symptoms, COVID-19, public interest, Google Trends, screening, breast cancer, symptoms, cancer, trend, mammography, monitoring

## Abstract

**Background:**

The COVID-19 pandemic has led to a decrease in cancer screening due to the redeployment of health care resources and public avoidance of health care facilities. Breast cancer is the most common cancer diagnosed in female individuals, with improved survival rates from early detection. An avoidance of screening, resulting in late detection, greatly affects survival and increases health care resource burden and costs.

**Objective:**

This study aimed to evaluate if a sustained decrease in public interest in screening occurred and to evaluate other search terms, and hence interest, associated with that.

**Methods:**

This study used Google Trends to analyze public interest in breast cancer screening and symptoms. We queried search data for 4 keyword terms (“mammogram,” “breast pain,” “breast lump,” and “nipple discharge”) from January 1, 2019, to January 1, 2022. The relative search frequency metric was used to assess interest in these terms, and related queries were retrieved for each keyword to evaluate trends in search patterns.

**Results:**

Despite an initial drastic drop in interest in mammography from March to April 2020, this quickly recovered by July 2020. After this period, alongside the recovery of interest in screening, there was a rapid increase in interest for arranging for mammography. Relative search frequencies of perceived breast cancer–related symptoms such as breast lump, nipple discharge, and breast pain remained stable. There was increase public interest in natural and alternative therapy of breast lumps despite the recovery of interest in mammography and breast biopsy. There was a significant correlation between search activity and Breast Cancer Awareness Month in October.

**Conclusions:**

Online search interest in breast cancer screening experienced a sharp decline at the beginning of the COVID-19 pandemic, with a subsequent return to baseline interest in arranging for mammography followed this short period of decreased interest.

## Introduction

The COVID-19 pandemic has led to a decrease in cancer screening due to the prioritization of health care resources toward COVID-19–related efforts and changes in health care–seeking patterns [1]. This may have resulted in delays in diagnosis and treatment [2,3], possibly adversely impacting oncologic outcomes.

This is particularly important in breast cancer as it is the world’s most prevalent cancer and the leading cause of cancer death in female individuals [4]. An important measure to reduce breast cancer mortality is the use of population screening by mammography, which aims to diagnose breast cancer at an earlier stage [5]. Female individuals diagnosed with breast cancer at an early stage have 5-year survival rates in excess of 90%, dropping to 85% after locoregional spread and 29% with distant disease [6]. With the World Health Organization (WHO) declaration of a global health emergency in January 2020, followed by the subsequent declaration of the COVID-19 pandemic in March 2020, there was a 74.3% reduction in public interest in mammograms compared to the prepandemic period [7].

In today’s technological era, it is common to seek health information on the internet to fill the gap between information one already has and what one seeks to know [8]: 72% of US adults reported seeking health information online, and 77% started with an internet search engine [9]. Google is the primary search engine and accounts for more than 90% of internet searches [10]. Online search interests for COVID-19–related issues peaked with increasing COVID-19 case numbers, which corresponds to the known phenomena of redirected health care resources [11]. The Google Trends [12] tool has been used to measure public interest in various oncological topics [13].

Google Trends has been shown to be a viable tool to understand, monitor, and even forecast information-seeking trends and public interest. It is an increasingly popular method for assessing population preferences in health research [14-16]. Google Trends provides a quick and easy way to access public interest in any topic across time and geographical location. It uses publicly available data, which allow studies to be transparent and easily reproducible. In addition, as data are available in real time, it solves issues with traditional and time-consuming survey methods [13].

Most existing literature on the effect of COVID-19 on cancer screening focused primarily on the medical implications, such as delays in diagnosis and treatment [7,17]. This study instead focused on public perception by analyzing search engine queries to assess if there had been a decrease in interest for breast cancer screening over time since the beginning of the pandemic and whether this has recovered to prepandemic levels. Additionally, this study aimed to observe the other terms that are searched alongside breast cancer screening. These may indicate new areas that need attention with regard to public health initiatives or education campaigns during such periods.

To achieve these objectives, trends in Google search volume were analyzed for mammography, breast self-examination, breast lumps, nipple discharge, and breast pain before and during the COVID-19 pandemic. In comparing these trends, we aimed to illustrate the pandemic’s effect on public interest in breast cancer screening and related symptoms.

## Methods

### Overview

A retrospective study of a publicly available query tool that aggregates data on Google search trends was conducted. Google Trends is a useful tool for tracking the frequency of search terms over time. It can be used to analyze changes in public interest or awareness of certain topics, including breast cancer screening. With these data, researchers and health professionals gain insight into how people are searching for information about breast cancer screening and where the gaps in their knowledge may be.

Using Google Trends, different search terms related to breast cancer screening may be compared. This allows users to understand the search term that has garnered more interest from the public over a period of time. In addition, comparisons can also be made between different geographic regions, time frames, and categories.

The data for any search term are normalized to the time and location of a query by the division of the total searches of the geography and time range it represented, to compare relative popularity. The relative popularity for any term is reported as a relative search frequency (RSF) from 0 to 100, with 100 representing the peak popularity of a term.

A search for a single term gathers results that include the specific word queried. Next, a search of multiple terms includes each word in any order. A search for a term in quotes obtains results that include the specific order of words queried. An alternative search strategy uses *topics*, a group of predefined terms that share the same concept in any language. For example, the topic “breast cancer” will include results for topics such as “brustkrebs,” which is “breast cancer” in German. Finally multiple queries can be searched concurrently to compare RSF across the terms (comparison) or individually to reflect the RSF of each individual term (individual search), which is more useful when comparing trends across terms in comparison to relative frequency.

Google Trends can also be used to evaluate related queries, which report on related search terms that users also search for alongside the index search terms. “Top” terms represent the most popular search terms scaled to the most commonly searched query as 100, and “rising” terms represent the queries with the biggest increase in search frequency during the requested time period.

On February 6, 2022, Google Trends was queried with keyword terms representing interest in breast cancer screening (“mammogram”) or breast cancer symptoms (“breast pain,” “breast lump,” and “nipple discharge”) as a comparison. “Mammogram” was selected to represent breast cancer screening as this is the standard modality for breast cancer screening. Breast cancer symptoms are common symptoms that patients with breast cancer may experience, or alternatively, patients with otherwise benign conditions may experience and thus require further investigation to rule out a breast malignancy. The 3 most common symptoms were included as search terms. In contrast to “mammogram,” the query “breast self-examination” does not require a medical provider visit and so may be less affected by COVID-19; thus, this was included separately as a search term to be analyzed. A worldwide search from January 1, 2019, to January 1, 2022, using the “all-categories” query category was conducted. January 1, 2019, was chosen as the start date to capture baseline interest, the “worldwide” setting was selected to capture search information worldwide, and the “all-categories” query category was chosen to assess interest in any context and to avoid any bias in filtering search results. Related queries were also retrieved for each of the key terms for the same time period. Further searches to compare peaks of terms were conducted as individual searches based on the results retrieved. Since searches included “how to cure breast lump naturally,” this was compared to the terms “lumpectomy” and “breast biopsy” as possible routine next steps in the management of a breast lump in contrast to natural treatment.

To assess whether a change in search volumes for “mammogram” was significant, we conducted time series forecasting using an autoregressive integrated moving average (ARIMA) model variant model allowing for seasonal variability (seasonal ARIMA). Expected search interest for “mammogram” from January 1, 2019, to January 1, 2022, was estimated using the seasonal ARIMA model based on the searches for “mammogram” during the 5 years prior to COVID-19 (from January 1, 2014, to January 1, 2019)

### Ethical Considerations

This study involved cross-sectional analysis of publicly available search engine metadata and does not use data on or involve individual human subjects; thus, it fulfills the criteria for institutional review board exemption.

## Results

**Figure 1:**
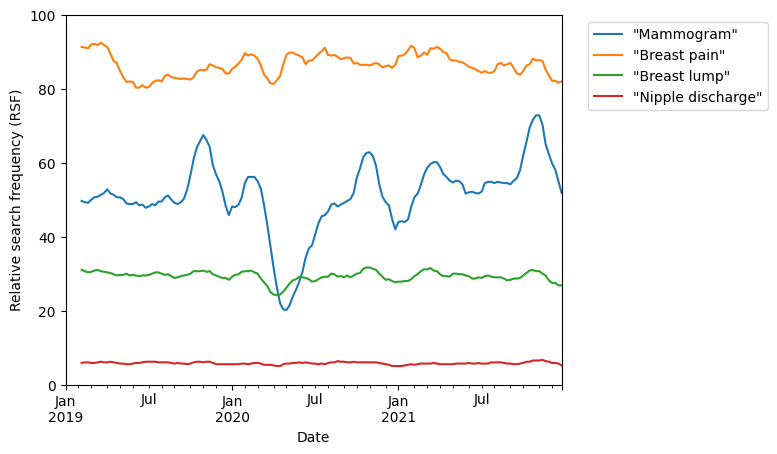
Online search interest in "mammogram" and breast cancer related symptoms ("breast pain," "breast lump," and "nipple discharge") during the time period from January 1, 2019, to January 1, 2022. Google Trends relative search frequency (RSF) is reported as a value from 0 to 100, with 100 representing peak popularity of the term over the time period.

**Figure 2:**
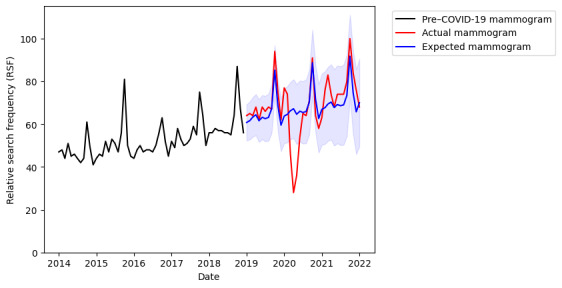
Online search interest in "mammogram" during the time period from January 1, 2014, to January 1, 2019 (pre–COVID-19 mammogram); actual search interest during the time period from January 1, 2019, to January 1, 2022 (actual mammogram); and expected search volume during the latter period predicted from the previous 5 years' worth of data (expected mammogram). Google Trends relative search frequency (RSF) is reported as a value from 0 to 100, with 100 representing peak popularity of the term over the time period as individual search. Shaded area represents 95% CI.

In the period from 2019 to 2022, a significant drop in searches for “mammogram” was found from March to April 2020 (Figure 1). This drop is significant compared to the expected search interest based on previous 5 years’ worth of search data (*P*<.001; Figure 2). This coincided with the start of the COVID-19 pandemic, announced at the WHO media briefing on March 11, 2020 [18], and the subsequent deferment of nonurgent elective cases and outpatient clinic appointments in response to this in many countries [19,20]. Searches for “mammogram” recovered to a pre–COVID-19 baseline by July 2020.

**Figure 3:**
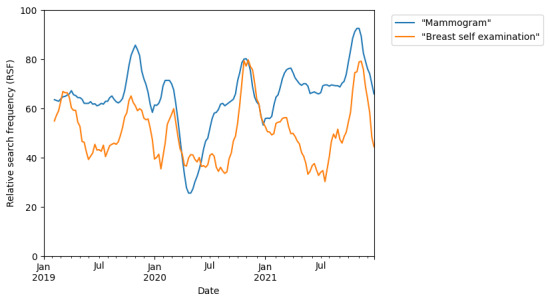
Online search interest in "mammogram" and "breast self examination" during the time period from January 1, 2019 to January 1, 2022. Google Trends relative search frequency (RSF) is reported as a value from 0 to 100, with 100 representing peak popularity of the term over the time period.

Notwithstanding the drop in searches for mammogram, interest in breast self-examination did not show any drop in comparison to baseline annual values for the same time period (Figure 3). Also seen in Figure 2 are the October peaks in interest for both mammogram and breast self-examination when the search frequency rises to 1.6-2 times of baseline, coinciding with the internationally designated *Breast Cancer Awareness Month* (BCAM) [21]. This is in contrast to searches for breast cancer symptoms that did not show any increased search volume coinciding with the October BCAM (Figure 1).

To further elucidate the type of information that is searched for in relation to mammogram or breast cancer symptoms, related search terms were assessed. The top and rising terms reported by Google Trends represent search terms that users also searched for alongside the index search terms, thereby giving an unbiased expanded and related search landscape.

The most common related search for mammogram was for basic information: “breast mammogram” and “mammogram screening” (100 and 54 RSF, respectively). This could be a result of individuals seeking information prior to consulting a health care professional for reasons such as obtaining information about disease symptoms, diagnosis, and treatment [22].

**Figure 4:**
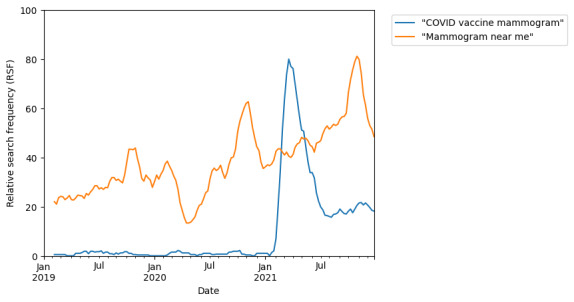
Online search interest in "covid vaccine mammogram" and "mammogram near me" during the time period from January 1, 2019 to January 1, 2022. Google Trends relative search frequency (RSF) is reported as a value from 0 to 100, with 100 representing peak popularity of the term over the time period.

**Figure 5:**
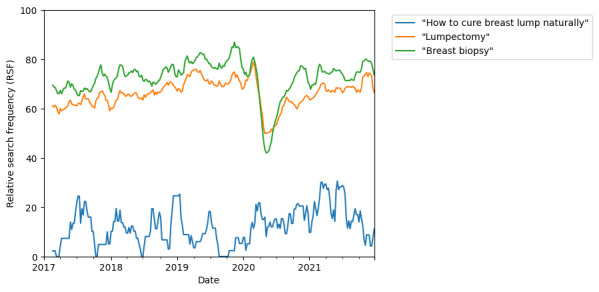
Online search interest for "how to cure breast lump naturally," "lumpectomy," and "breast biopsy" between January 1, 2017, and January 1, 2022. Google Trends relative search frequency (RSF) is reported as a value from 0 to 100, with 100 representing peak popularity of the term over the time period.

During the period from 2019 to 2022, there was an increased interest in the relationship of the COVID-19 vaccine and mammography, the timing of mammogram after COVID-19 vaccination (“covid vaccine mammogram” and “mammogram after covid vaccine”), as well as an increased interest in arranging for mammograms (“mammogram screening near me”; Table 1). When interest in arranging for mammography (“mammogram near me”) was analyzed, we saw that not only did this recover after the initial dip from March to June 2020, but it also exceeded pre–COVID-19 levels. An increased peak size coinciding with the October BRAM (Figure 4) was also seen. Although a relationship between COVID-19 vaccination and mammogram emerged during this time period, after an initial peak at the start of 2021, this quickly diminished in frequency as a search term (Figure 4). An increase in searches for natural and nonsurgical treatment of breast lumps (+170% and +120%, respectively; Table 2) was also observed, which coincided with the start of the COVID-19 pandemic and a drop in interest in lumpectomy and breast biopsy from March to June 2020 (Figure 5).

**Table 1:** Search terms associated with “mammogram” from January 1, 2019, to January 1, 2022. Relative search frequency (RSF) for “top” terms is reported as a value from 0 to 100, with 100 representing peak popularity of the term over the time period. “Change over time” for rising terms represents the largest increase in search frequency over the aggregated time period.

Term		Value
**Top, RSF**		
	breast mammogram	100
	mammogram screening	54
	mammogram cancer	53
	mammogram near me	41
	breast cancer	39
	breast cancer mammogram	39
	what is mammogram	32
	mammogram age	32
	ultrasound	29
	ultrasound mammogram	29
	diagnostic mammogram	24
	3d mammogram	24
	mammogram icd 10	23
	what is a mammogram	18
	mammogram cost	16
**Rising, change over time**		
	covid vaccine mammogram	173,100
	covid vaccine and mammogram	53,800
	mammogram after covid vaccine	53,550
	mammogram screening near me	450
	focal asymmetry on 3d mammogram	350
	breast mammogram near me	250
	mammogram near me	180
	mammogram screening icd-10	160
	free mammogram near me	150
	lenox hill radiology	150
	schedule a mammogram near me	150
	mammogram test near me	130
	mobile mammogram near me	120
	obgyn near me	120
	private mammogram	120

**Table 2:** Search terms associated with “breast lump” from January 1, 2019, to January 1, 2022. Relative search frequency (RSF0 for “top” terms is reported as a value from 0 to 100, with 100 representing peak popularity of the term over the time period. “Change over time” for rising terms represents the largest increase in search frequency over the aggregated time period.

Term		Value
**Top, RSF**		
	lump in breast	100
	cancer breast lump	42
	breast cancer	42
	lump on breast	35
	breast lump pain	21
	breast pain	21
	painful lump breast	19
	painful breast	19
	lump in the breast	14
	lump under breast	14
**Rising,** **change over time**		
	breast lump when to worry	400
	lump under breast near ribs	350
	how to cure breast lump naturally	170
	lump on breast bone pictures	160
	lump in breast meaning	120
	ache in breast no lump	120
	how to cure breast lump without surgery	120
	which doctor to consult for breast lump	100
	left breast lump icd 10	100
	pain in left breast	90

## Discussion

### Principal Findings

The purpose of this study was to evaluate the impact of the COVID-19 pandemic on public interest in breast cancer screening and its subsequent recovery. Despite an initial drop in interest in mammography from March to April 2020, it quickly recovered by July 2020. After this period, alongside the recovery of interest in screening, there was a rapid increase in interest for arranging for mammography as indicated by searches for “mammogram near me.”

Previous studies have documented a decrease in cancer screening and diagnosis during the COVID-19 pandemic but not individual’s interest in screening during the crisis [23-25]. The initial dip in search volume could be explained by the postponement of elective visits during the first wave of the pandemic, as searches are usually prompted by upcoming visits and discussions with providers [23,24], or a reluctance in seeking medical attention due to the fear of contracting COVID-19 in the health care setting. As a result of reduced health care contact, newly diagnosed cancer rates declined by 46.4% after the start of the pandemic [26].

Despite the drop in screening, we demonstrated consistent levels of interest in breast cancer–related symptoms and breast self-examination during this period. During times of reduced health care contact, patients continued to use the internet to search for their symptoms. This is concerning given that the use of “Dr. Google” has been linked to increased self-medication and the decision not to see a medical professional [27]. This is reflected in our results showing increased interest in natural treatment of breast lumps, which remains sustained despite the recovery of interest in mammography and breast biopsy.

Our study shows a correlation between health campaign (BCAM) and search behaviors. This result supports previous studies that suggest that infoveillance can measure the success of a campaign in driving information-seeking behaviors in a population [28,29].

### Limitations

There are a few limitations to our research. First, there is a potential overrepresentation of younger, more technologically savvy internet users actively searching for health-related terms. Second, data from Google Trends may not represent a portion of the public who do not have internet access, for example, in countries lacking the infrastructure and technology or with lower socioeconomic status. Third, there may be an overrepresentation of English-speaking users who tend to use Google as a primary search engine. For example, instead of Google, Baidu is the top search engine in China with an 83.46% market share [30]. Lastly, even though results indicate general trends, it does not highlight specific subgroups or give information about the demographics of users who could have a higher share in search volumes.

### Comparison With Prior Work

Similar prior studies have previously documented a decrease in individuals’ interest in screening during the first peak of the COVID-19 pandemic [7]. We have demonstrated that there does not seem to be lasting adverse effects on public interest in breast cancer screening as interest in mammography and arranging for mammograms returned to or exceeded the pre–COVID-19 level. This recovery may have been aided by BCAM. There are similar levels of peak interest in mammography and breast self-examination during the October BCAM despite the ongoing pandemic.

### Conclusions

In conclusion, online search interest in breast cancer screening experienced a sharp decline at the beginning of the COVID-19 pandemic with a subsequent return to baseline interest in arranging for mammography following this short period of decreased interest.

Our study shows that despite concerns about the impact of the COVID-19 pandemic on breast cancer screening, interest in mammography quickly recovered. This has implications for health care providers leveraging this recovery to encourage more individuals to get screened, especially among those who may have delayed their mammogram due to the pandemic and for health service resource allocation to respond to this rapid recovery in interest.

Additionally, the study highlights the importance of monitoring changes in search behaviors related to health care during a crisis, as it may reflect changes in health care–seeking behaviors in the general public.

Future work could investigate whether the pandemic had a differential impact on cancer screening rates and outcomes among different populations, including racial and ethnic minority groups, rural populations, and low-income individuals. It would also be important to assess if the COVID-19 pandemic had a long-term impact on cancer outcomes including delayed diagnoses and increased morbidity and mortality.

## Abbreviations

ARIMA: autoregressive integrated moving average
BCAM: Breast Cancer Awareness Month
RSF: relative search frequency
WHO: World Health Organization

## Data Availability

Google Trends data are available to all users free of charge [12].
